# Decoding sex differences in human immunity through systems immunology

**DOI:** 10.1093/oxfimm/iqaf006

**Published:** 2025-07-04

**Authors:** Joan Escrivà-Font, Tianze Cao, Camila Rosat Consiglio

**Affiliations:** Systems Immunology Lab, Division of Molecular Hematology, Department of Laboratory Medicine, Lund Stem Cell Center, Lund University, Lund, 221 84, Sweden; Systems Immunology Lab, Division of Molecular Hematology, Department of Laboratory Medicine, Lund Stem Cell Center, Lund University, Lund, 221 84, Sweden; Systems Immunology Lab, Division of Molecular Hematology, Department of Laboratory Medicine, Lund Stem Cell Center, Lund University, Lund, 221 84, Sweden

**Keywords:** systems immunology, sex differences in human immunity, sex hormones, biological sex

## Abstract

Immune function varies widely across humans. Biological sex is a key factor underlying human immune variability, with men presenting with more severe infections and increased cancer rates, while women exhibit higher vaccine responses and prevalence of autoimmunity. Intrinsic biological sex differences arise from varying contributions of chromosomal sex, and sex hormone sensing and downstream signaling to different cell types. This complex regulation presents a unique opportunity for the exploration of human immune sex differences using systems-level methods of investigation. Here we analyze the current literature and the applications of systems immunology in elucidating the immune sex differences in humans. We examine mechanisms of biological sex modulation of human immunity via sex chromosomes, and particularly emphasize the role of sex hormones. We then focus on how systems immunology has been advancing our understanding of how sex impacts the healthy immune system at steady state, ranging from cell composition, transcriptomics, epigenomics, metabolomics, spatial and cell-cell interactions, to plasma proteomics. We also examine systems-level applications to investigating sex differences upon immune perturbations and give an overview of key future directions for the field. Systems immunology provides a powerful framework to decode biological sex-regulated pathways in immunity, paving the way for more precise, sex-informed therapeutic interventions to address sex differences in immune-related conditions.

## Introduction

Immune function varies widely in humans. The combination of unique extrinsic environmental exposures and intrinsic physiological processes results in stark differences in infection and immune-related diseases across individuals. The COVID-19 pandemic underscored this immune function variability, with SARS-CoV-2 exposure leading to outcomes ranging from asymptomatic to severe disease and post-acute infection syndromes [1]. While a variety of identified risk factors have been identified for immune-related diseases, it remains a major challenge to predict person-specific outcomes prior to immune insults such as viral exposure. This stems from both the absence of simple, reliable metrics to assess immune function in the clinics [2] and a limited holistic understanding of mechanisms driving human immune variability. Advances in experimental and computational immunology approaches are now enabling the systematic profiling of the human immune system, paving the way for a more refined understanding of human immune function and for more tailored approaches to prevent, predict, and treat immune-related diseases.

Traditionally, immunology research has employed reductionist approaches to characterize cellular and molecular pathways underlying human immune function. While such frameworks allow for mechanistic investigation in high depth, they lack the ability to profile the human immune system in a holistic manner. The immune system is highly complex: at the cellular and molecular levels, a wide range of immune cell types arise from diverse ontogenies and circulate throughout the body and/or reside in tissues. Immune subsets are equipped with varied functions and states and interact with and co-regulate one another at steady state. While local factors regulate immunity at the tissue level, the integrated response to immune stimuli requires coordinated whole-body communication between the site of initial insult and other tissues. Thus, variation at molecular, cellular, and systemic levels of immune regulation gives rise to heterogeneity in human immune responses. Originating from a systems biology framework, systems immunology is an innovative approach that aims to understand the complexity of the immune system and its components. Systems immunology combines high-throughput experimental methods with advanced computational analyses to unveil complex immune interactions and underlying determinants of immune function.

An important application of systems immunology lies in dissecting how intrinsic and extrinsic factors shape immune variability. Among intrinsic factors, biological sex emerges as a major regulator of immune function, driving significant differences in susceptibility and severity of infection and immune-related diseases. Women generally display heightened responses to vaccines and have increased prevalence of autoimmunity, while men mount less robust immune responses and show higher infection severity [3]. Systems immunology offers a holistic framework to dissect biological sex-regulated pathways in immunity and advance the development of tailored interventions for sex differences in immune-related conditions. In this review, we analyze the current literature and the applications of systems immunology in elucidating the immune sex differences in humans.

## Biological sex regulates human immunity

Biological sex differences are reported in infections [4,5], autoimmunity [6,7], inflammatory diseases [8], cancer [9], transplantation [10–12], and vaccination [13,14]. Acute and chronic infections are generally more severe and lead to higher mortality in males [15], including bacterial (e.g. *Mycobacterium tuberculosis*) [4], tropical parasitic [16], and viral (e.g. SARS-CoV-2) [17] infections. Conversely, a female bias is evident in post-acute infection syndromes [18], a series of symptoms persisting after acute infection, such as long COVID (LC) following SARS-CoV-2 infection [19,20]. Autoimmune disorders, such as multiple sclerosis (MS), systemic lupus erythematosus (SLE), and rheumatoid arthritis (RA), disproportionately affect females, with 80% of people suffering from autoimmune disorders being females [21]. Biological sex is also an important factor in transplantation success [10,11], with males receiving liver, kidney, and heart transplants from female donors showing worse outcomes [10, 22]. Female-to-male bone marrow transplantation shows a higher risk of acute graft-versus-host disease, and this risk is further accentuated for grafts obtained from female donors that were previously pregnant [11]. Moreover, females also display heightened responses to vaccines—with half a dose of influenza vaccination producing the same antibody titers as a full dose in males [13,14]. In addition, sex differences have been reported in CAR-T cell therapy [23] and checkpoint inhibitor therapy for cancer [24]. Overall, studies strongly suggest that females mount more robust immune responses compared to males, significantly affecting disease development and progression. Such striking immune sex differences are reported across many species, and have been reviewed elsewhere for fruit flies [25], mice [3, 26, 27], primates [28], and across species [29,30]. Here we focus on sex differences in the human immune system at different levels of biological organization, including cell composition, cell states, local interactions among immune cells, and dynamics upon perturbation.

Human immune sex differences originate from both biological sex and gender effects. Gender refers to a social construct that encompasses gender roles and other behaviors that influence how men, women, and others expose themselves to infections, seek healthcare, etc. Gender differences impact care-seeking, care quality, treatment adherence, and exposure to pathogens. In contrast, biological sex relates to physiological characteristics like sex chromosomes, sex hormones, genitals and gonads, and secondary sexual characteristics. Herein we focus on biological sex’s effects on immunity and use the terms male and female throughout the text, though we acknowledge that gender alone and its interaction with sex also contribute to the observed epidemiological sex differences.

### Chromosomal sex

Human females present two X chromosomes, while males possess an X and a Y chromosome. The X chromosome contains a higher number of genes compared to the Y chromosome, where most genes are related to sex determination and development. Of note, around 54 genes found in the X chromosome have immune-related functions [31]. X-linked genes are generally expressed in both sexes, while Y genes are only expressed in males. X chromosome inactivation (XCI) mediates gene dosage compensation and occurs during human embryonic development in females, suppressing expression from one of the X chromosomes at random [32]. Canonical XCI is maintained through *XIST* RNA expression in most cells, which binds to the inactivated X chromosome to repress gene expression. Conversely, non-canonical XCI maintenance has been observed for cells that maintain XCI while lacking *XIST* RNA expression [33], such as the case of peripheral blood B and T cells [31]. Yet, as many as 20% of X chromosome genes escape XCI, with this percentage being highly dependent on cell identity and functional state [34]. For instance, the viral sensor *TLR7* escapes XCI and shows higher expression in human B cells, plasmacytoid dendritic cells (pDCs), and monocytes from females, especially in female-biased autoimmune diseases such as SLE [35]. Females also display biallelic expression of *CD40LG* in T cells, which has also been associated with SLE [33, 36]. Moreover, XCI escapee genes can show elevated expression in female cells (e.g. *DDX3X*, *KDM6A*), while many do not exhibit any sex bias (e.g. *IKBG*, *USP9X*) [37]. Adding to the complexity of X chromosome gene expression, XCI is typically regarded as a random process, resulting in a mosaic pattern of cells where either the maternal or the paternal X chromosome is inactivated [32]. However, skewed XCI is relatively common in females [38], and complete XCI skewing—where the same parental X chromosome is inactivated across all cells—has also been observed in the developing thymic T cells of a pediatric donor [37]. Finally, sex chromosome numbers can deviate from the binary XX and XY. Disorders of sexual development (DSDs) are characterized by the presence of different combinations of sexual chromosomes, such as Turner (X0) or Klinefelter syndrome (XXY). Such differences in X chromosome numbers affect gene expression of X-linked genes *TLR7* and *CD40LG* in human peripheral blood mononuclear cells (PBMC) [39], and have also been linked to higher rates of autoimmune disease, most likely due to gene dosage alterations [40, 41].

The Y chromosome also shows a role in human immune function [42]. For instance, some Y haplogroups exhibit faster progression of HIV to AIDS, and higher mortality [43], potentially relating to the *DDX3X* gene, which is reported to be a key factor of HIV-1 replication [44]. The Y chromosome also contains specific proteins, such as antigen (H-Y) that can be immunogenic following transplantation of male donor kidneys into female recipients, leading to a worse prognosis [10, 22]. Moreover, the frequency of somatic loss of the Y chromosome in immune cells increases with age and has been identified as a risk factor for severe COVID-19 [45], Alzheimer’s, and prostate cancer [46].

### Sex hormones

Sex hormones play a central role in regulating physiological processes, including the development and function of the immune system. Females and males display striking differences in sex steroid production, circulating levels, and signaling over the course of life. Generally, males have higher concentration of androgens in circulation, such as testosterone, its more potent form dihydrotestosterone (DHT), and androstenedione, while females show higher levels of estrogens (estrone, estradiol, and estriol) and progestins (progesterone) during reproductive age [47].

#### Physiology of sex hormones

**Figure 1.**
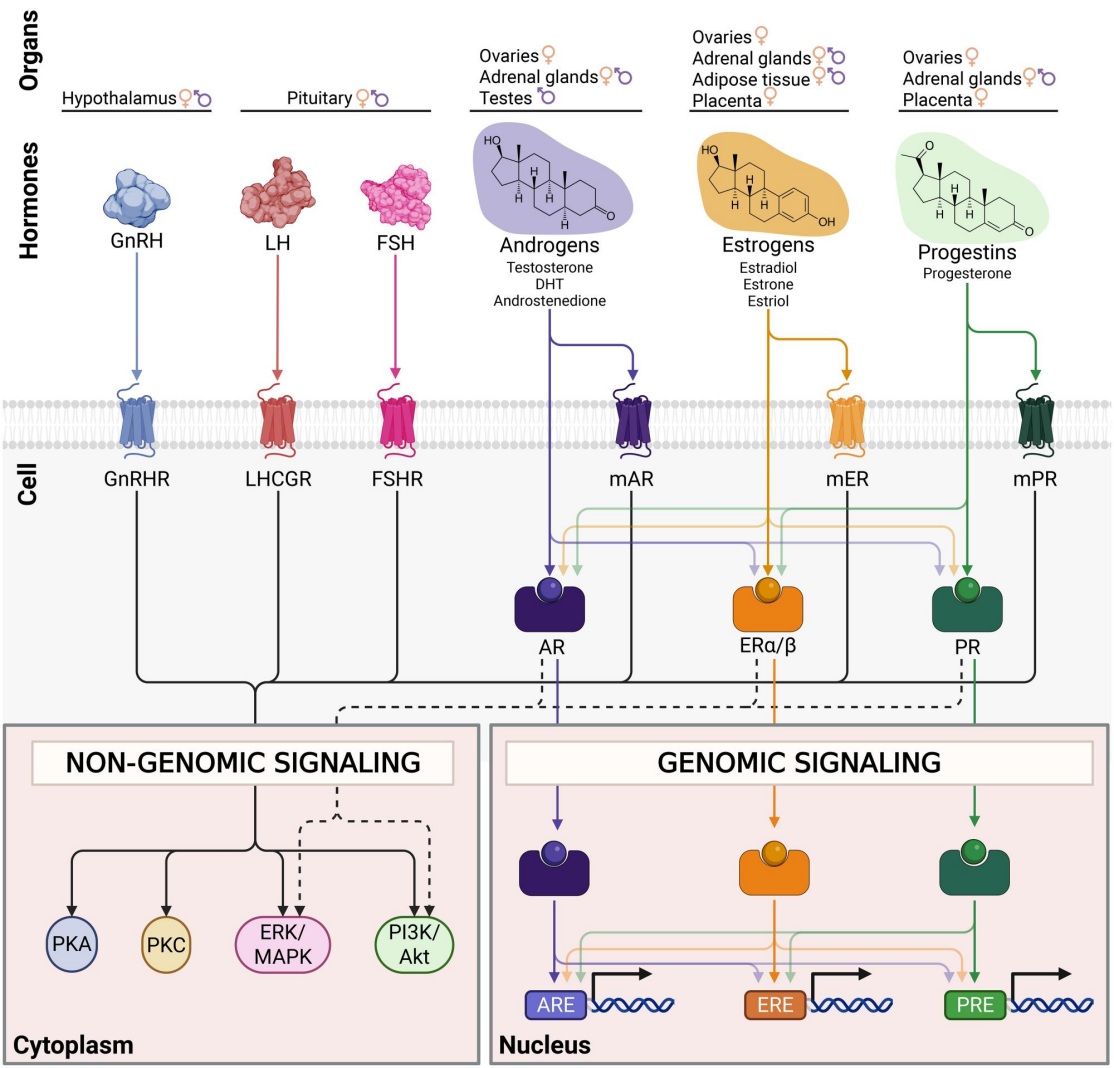
Overview of signaling pathways for sex hormones and upstream regulators. Interaction of hypothalamic–pituitary–gonadal axis protein hormones and steroid hormones with their receptors, highlighting their tissue sources, cognate receptors, downstream non-genomic and genomic signaling cascades. The hypothalamus-derived GnRH acts via the GPCR GnRHR triggering non-genomic signaling pathways. Pituitary-derived LH and FSH bind to their respective GPCRs, LHCGR and FSHR, activating similar non-genomic signaling cascades. Androgens, such as testosterone, DHT, and androstenedione, act through both membrane androgen receptors (mAR) and androgen receptors (AR). mAR is a GPCR that mediates non-genomic signaling, while the nuclear receptor AR initiates both non-genomic signaling, and genomic signaling by binding AREs in the nucleus. Estrogens, including estradiol, estrone, and estriol, act through both membrane estrogen receptors (mER) and nuclear ERα and ERβ. mER mediates non-genomic signaling, while nuclear receptors ERα/ERβ activate both non-genomic signaling, and genomic signaling by binding EREs. Progestins, like progesterone, act through membrane progesterone receptors (mPR), which induce non-genomic signaling, and nuclear PR, which initiates both non-genomic signaling, and genomic signaling pathways by PRE binding. Androgen, estrogen, and progestin signaling exhibit significant crosstalk. Sex hormones bind at low affinity to non-cognate sex hormone receptors, and sex hormone nuclear receptors can interact with various hormone response elements and cooperate or antagonize each other to activate or repress gene expression. Light-colored arrows indicate lower binding affinity. Intracellular signaling cascades depicted are derived from immune cells (GnRH, mER) [48, 49, 50], testicular Sertoli cells and ovarian granulosa (FSHR) [51], or cell lines derived from ovarian and testicular (LHCGR) [52], prostate (mAR) [53], breast (mPR) [54] or diverse types of cancer (AR, ERα/β, PR) [55]. AR: androgen receptor, ARE: androgen response element, ER: estrogen receptor, ERE: estrogen response element, ERK/MAPK: extracellular signal-regulated kinase/mitogen-activated protein kinase, FSH: follicle-stimulating hormone, FSHR: FSH receptor, GnRH: gonadotropin-releasing hormone, GnRHR: GnRH receptor, LH: luteinizing hormone, LHCGR: LH/choriogonadotropin receptor, mAR: membrane-bound AR, mER: membrane-bound ER, mPR: membrane-bound PR, Pi3K/Akt: phosphoinositide 3-kinase/protein kinase B, PKA: protein kinase A, PKC: protein kinase C, PR: progesterone receptor, PRE: progesterone response element. Created in BioRender. Escrivà Font, J. (2025) https://BioRender.com/ejhfg27.

Sex hormone levels are mainly controlled by the hypothalamic-pituitary-gonadal axis (HPG). Hypothalamus-derived gonadotropin-releasing hormone (GnRH) stimulates pituitary gland production of follicle-stimulating hormone (FSH) and luteinizing hormone (LH), which are gonadotropins that control the production of steroid hormones (Fig. 1). The site of androgen production differs between the sexes, being mostly produced in the testes in males, while female androgen precursors are generated in the ovaries and adrenal cortex and then transformed to testosterone in the periphery [56]. In females at reproductive age, the most potent estrogen is estradiol, which is predominantly produced by the ovaries and by the placenta during pregnancy [57, 58]. Following menopause, estrogen levels significantly drop and mainly derive from adipose tissue aromatization of circulating steroids [56]. Adipose tissue is also the major estrogen source in males [57, 58]. Similar to estrogens, progesterone is primarily produced by the ovaries and by the placenta during pregnancy in females at reproductive age, and by the adrenal cortex after menopause in females and throughout life in males [59].

#### Signaling of sex hormones

GnRH, gonadotropins, and sex steroids exert their actions via intracellular or membrane-bound receptors (Fig. 1). Androgens, estrogens, and progestins bind to their cognate intracellular nuclear receptors (AR, ERα & ERβ, PR, respectively), which act as transcription factors that quickly modulate gene expression in immune cells. In addition, sex hormones show “ligand promiscuity” and can bind to other non-cognate nuclear receptors, though at low, varying affinities [60, 61]. Of note, AR, ERα, and ERβ are widely expressed across human blood immune cell types, including neutrophils, dendritic cells, monocytes and macrophages, T cells and B cells, while human bone marrow immune cell progenitors also express AR, ERα, and PR [62]. Overall, PR expression seems to be more restricted, though it has also been detected in uterine NK cells [63]. Yet, it remains unclear the extent of sex hormone receptor expression across human tissue resident immune cells. Sex steroid nuclear receptors bind to their corresponding androgen, estrogen, and progesterone response elements (i.e. ARE, ERE, PRE), which are widely present across the human genome (Fig. 1). While immune cells express sex hormone receptors, sex hormone response genes and related pathways have been mostly described for cells that highly express sex hormone receptors, such as cancer cells. In fact, current sex hormone response gene sets (MSigDB [64, 65]), regions with nuclear receptor motifs (MSigDB [64, 65]), and direct sex hormone receptor-DNA binding experimental evidence (CistromeDB [66, 67] and TFLink [68]) are predominantly derived from cancer cell lines. Although a comprehensive list of direct targets of sex hormone nuclear receptors is still missing for immune cells, we highlight selected sex hormone receptor target genes in human immune cells from chromatin immunoprecipitation (ChIP) evidence (Table 1). Such targets include, for instance, *IL1B*, *FOXP3*, and *CCR7*, and highlight a role for direct sex hormone receptor regulation of T cell differentiation and chemokine signaling. Moreover, crosstalk among nuclear hormone receptors has been reported, including DNA binding in other hormone response elements, but also cooperative activation or repression, and antagonistic effects of target genes [75–78], but their relevance in immune cells is not fully resolved. In sum, sex steroids can induce long-lasting effects by regulating the epigenome via nuclear sex hormone receptors [79]. Alternatively, nuclear receptors can also exert their effects through rapid non-genomic signaling, involving kinases such as PI3K/Akt and ERK/MAPK pathways [55] (Fig. 1). Finally, sex hormones can also induce intracellular signaling pathways via membrane-bound G protein-coupled receptors (GPCR) AR, ER, and PR [50, 80]. When activated, GPCRs typically exert their effects through sequential activation of kinases such as MAPK, PI3K/Akt, PKA, or PKC (Fig. 1), though evidence regarding the role of this signaling in human immune cells is relatively limited.

**Table 1.** Selected sex hormone receptor target genes in human immune cells from ChIP-seq and ChIP-PCR data

Receptor	Target gene	Immune cell subset	Biological context	Reference
AR	*IL1B*	Tumor-associated macrophages	Prostate cancer patients (chr. XY)	Ref. [69]
	*TREM-1, CCL2, CCL3, CCL7, CCL13, IL8, IL10*	PMA-activated THP-1 cells	Cell line from a leukemia patient (chr. XY)	Ref. [70]
	*FOXP3*	Peripheral blood regulatory T cells	Healthy patients (chr. XX and XY)	Ref. [71]
ERα	*FOXP3*	Regulatory T cells	Cervical cancer patients (chr. XX)	Ref. [72]
	*CD16*	PMA-activated THP-1 cells	Cell line from a leukemia patient (chr. XY)	Ref. [73]
ERβ	*CXCR4, CCR7, IL10RA*	Mantel-cell lymphoma cells	Cell line from a B cell lymphoma patient (chr. XY)	Ref. [74]

The upstream regulators of sex steroids, GnRH, FSH, and LH, also signal through membrane-bound GPCRs (Fig. 1). GnRH receptor expression has been reported in immune cell subsets, such as blood CD4^+^ T cells, B cells, plasma cells, and bone marrow progenitors [48, 62, 81], while bone marrow and thymic immune progenitors express FSH and LH receptors [62, 82]. However, the extent of the interplay between the HPG axis and the immune system has not been studied in a comprehensive way to date [83]. Thus, the complexity of biological sex regulators in human immunity arises from the varying contribution of chromosomal sex, and sex hormone sensing and downstream signaling to individual cell types.

#### Sex hormone levels across life

**Figure 2.**
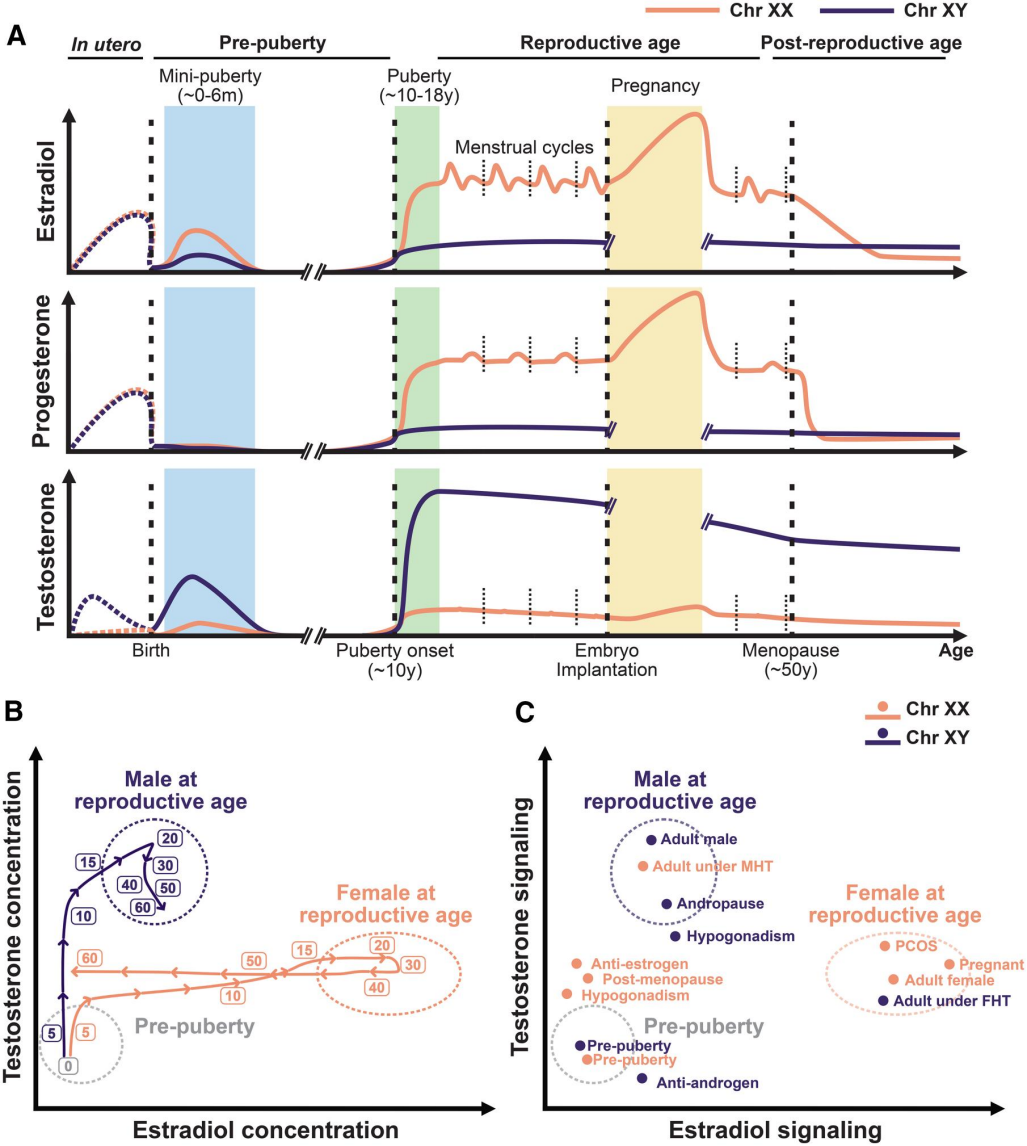
Sex hormones across lifespan in humans. **A**. Estradiol, progesterone, and testosterone levels across different endocrinological phases of life for individuals with XX and XY chromosomal sex. *In utero* levels are inferred from maternal serum and amniotic fluid levels (dashed curves), whereas post-natal values are derived from blood measurements (solid curves). Dashed vertical lines indicate notable hormone transitions, including birth, puberty onset, embryo implantation, and menopause. Short, dotted lines delineate each menstrual cycle. Ref [47, 86, 87, 298–300]. **B**. Trajectories over the course of life for testosterone and estradiol blood concentrations in individuals with XX and XY chromosomal sex. Both sexes start at low circulating sex hormone concentrations at birth and diverge during puberty. Males show a steady testosterone decline during andropause, while female menopause shows faster kinetics in estradiol concentration reduction. Variability in hormone concentrations is depicted with dotted circles. Ref [47]. **C**. Landscape of testosterone and estrogen signaling for different human cohorts across life stages, sex-hormone altering conditions, and pharmacological modulations. Comparison of immune measurements among these groups can enable investigation of sex chromosomes and sex hormone modulation of human immune function. PCOS: Polycystic ovarian syndrome. MHT: Masculinizing hormone therapy. FHT: Feminizing hormone therapy. Orange denotes individuals with XX chromosomal sex, and purple denotes individuals with XY chromosomal sex.

The circulating levels of sex hormones are highly dynamic across life, with three distinct phases throughout the human lifespan (Fig. 2A-B). The first exposure to sex hormones takes place during fetal development. Maternal- and placental-derived sex steroids are metabolized before reaching the fetus [84–86]. Fetal testes start producing testosterone at around 10 weeks of gestation and lead to masculinization of the male fetus [87]. Birth is followed by mini-puberty at 0–6 months, a period when gonadotropins rise in the neonate due to the activation of the HPG axis and lead to increased concentrations of testosterone in males and estradiol in females [88]. However, these levels quickly drop and remain minimal during childhood [88]. During puberty, the reactivation of the HPG axis allows the rise of sex hormones [89] (Fig. 2B). Interestingly, it is during puberty that the incidence of sex differences in immune-related diseases like SLE or allergic asthma significantly diverges between the sexes [90].

Testosterone remains relatively stable through male adulthood, while the concentrations of estrogens and progesterone vary throughout the female reproductive cycle (menstrual cycle, pregnancy, and menopause), which are associated with changes in disease risk and severity in females [91, 92]. The female menstrual cycle begins with the follicular phase, where a drop in progesterone levels is followed by hypothalamic release of GnRH, which stimulates the anterior pituitary to secrete FSH and LH. FSH stimulates the ovaries to produce estrogen. At mid-menstrual cycle, estrogen produces a sudden surge in LH levels that triggers ovulation. The luteal phase follows, where progesterone is secreted by the ovarian corpus luteum. If no fertilization takes place, both progesterone and estrogen levels drop, leading to menstruation, and a new cycle starts. Interestingly, the severity of symptoms of chronic autoimmune diseases has been reported to vary along the menstrual cycle. For instance, SLE and MS symptoms worsen during the luteal phase and menstruation [92–95], whereas RA has been shown to worsen during the follicular phase [93, 96, 97]. Conversely, if fertilization occurs, the cycle is interrupted with the implantation of the embryo starting pregnancy. During early pregnancy, secretion of human chorionic gonadotropin (hCG) by the embryo maintains the corpus luteum production of progesterone and estrogen, reaching considerably higher concentrations than a typical menstrual cycle. As pregnancy progresses, estrogen and progesterone levels continue to rise steadily, supporting fetal growth and preparing the body for labor. Autoimmunity symptoms improve during pregnancy for MS and RA [91, 98] and worsen again after delivery, while SLE flares are more frequent in pregnant individuals [91, 98].

Significant immune changes also occur later in life, where estrogen drops sharply during female menopause and reach levels comparable to those found in males [99] (Fig. 2B). This is coupled with changes in symptoms in some autoimmune diseases such as less flares in SLE, or increased severity of MS [91]. Conversely, testosterone levels steadily decline in males—around 1% per year [100]—during a process known as andropause or late-onset hypogonadism. In addition, about 20% of males over the age of 70 exhibit significantly low testosterone levels [101]. The general decline in male testosterone levels has been associated with an increase in the diagnosis of MS with age [102].

#### Altered sex hormone dynamics

Multiple conditions that affect hormone levels and signaling are associated with immune dysfunction (Fig. 2C). Higher concentration of testosterone in the blood is a hallmark of polycystic ovarian syndrome (PCOS) in women, and PCOS has been linked to an increased pro-inflammatory state [103]. Conversely, hypogonadism [104] is characterized by diminished gonadal sex hormone production in males and females. Males with untreated hypogonadism (without exogenous testosterone supplementation) show a higher risk of RA and SLE [105]. Female hypogonadism is often associated with DSDs like Turner syndrome (X0) [106], which present with increased risk of autoimmune disease, such as juvenile RA and Hashimoto’s thyroiditis [107]. Other intersex conditions like the androgen insensitivity syndrome, caused by a genetic defect in the X-linked AR gene, have been linked to increased risk of autoimmune diseases like Sjögren's syndrome [108] and celiac disease [109].

Pharmacological modulation of sex hormone levels and signaling also impacts immune function (Fig. 2C). For instance, the use of oral contraceptives, usually containing estrogen and/or progestin, has been associated with increased risk of autoimmunity [110]. Moreover, transgender women receiving exogenous estrogen therapy coupled with GnRH agonist for feminizing hormone therapy (FHT) [111] show increased risk of developing MS [112]. Yet, the long-term consequences of FHT and masculinizing hormone therapy (MHT) [113] on immune responses and immune-related diseases remain widely unknown. In addition, antiandrogens and androgen deprivation therapy (ADT) are routinely used for prostate cancer treatment [114], while antiestrogens target ER^+^ breast tumors, both of which have been reported to have additional immunomodulatory effects [115]. For instance, ADT enhances CD8^+^ T cell cytotoxicity [116], while antiestrogens increase NK cell cytotoxicity [117] and activate neutrophil proinflammatory pathways [118]. Because of such immunomodulatory properties, pharmacological modulation of sex hormones has also been harnessed in clinical trials of immune-related diseases. However, results are variable, with antiandrogen treatment aimed at dampening inflammatory responses not showing improvement in severe COVID-19 recovery [119], while different sex hormone modulation strategies show varying effects in MS clinical symptoms [120].

Overall, biological sex shapes immunity through the interplay of sex chromosomes, sex hormone signaling, and their effects on cell types across lifespan, presenting significant challenges for human immune investigation. The complex diversity in genetic and sex hormone signaling across human cohorts, including transgender and intersex individuals, presents as a unique opportunity for exploration of human immune sex differences using systems-level methods of investigation (Fig. 2B-C).

## Investigating immune sex differences through systems immunology

**Figure 3.**
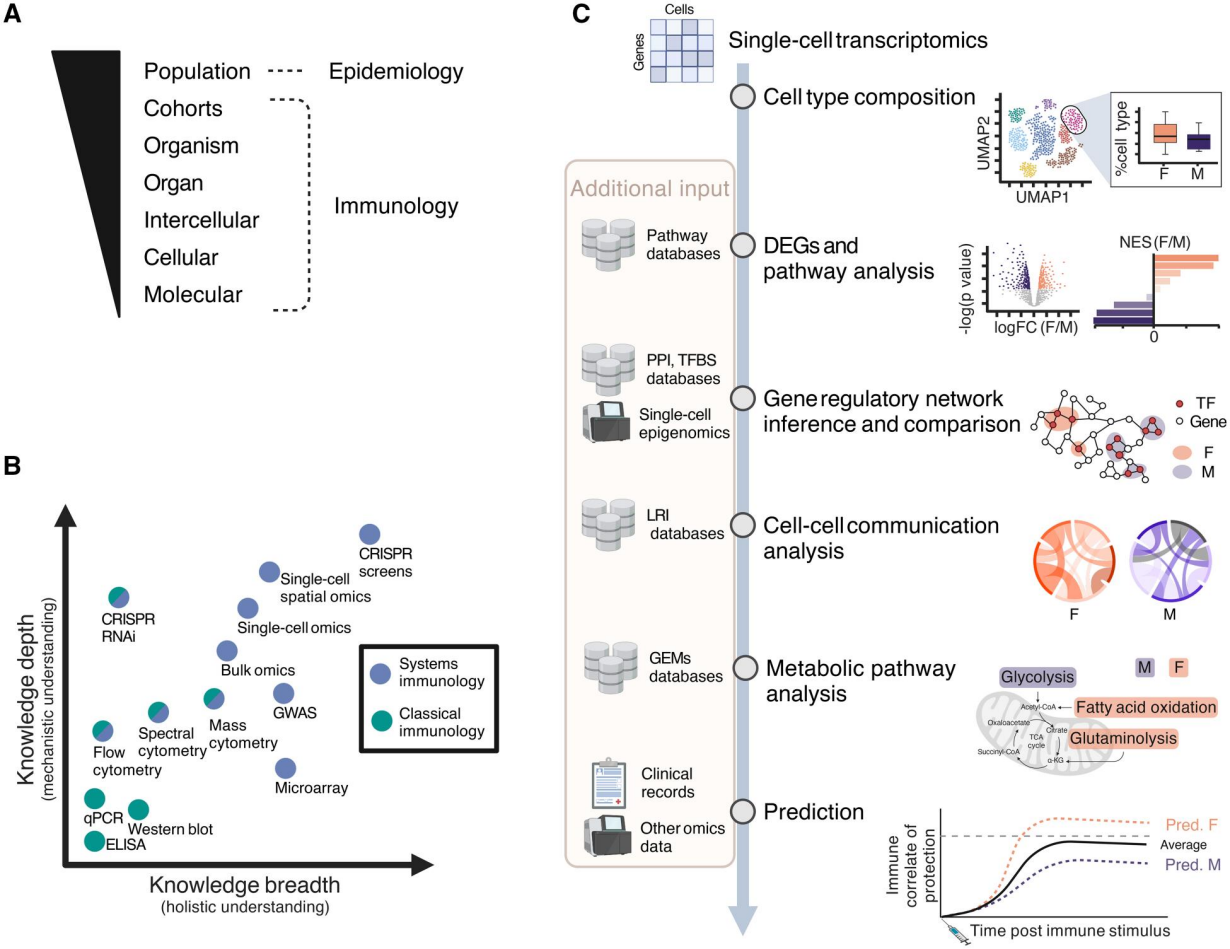
Multiscale immunology investigation via system immunology approaches. **A**. Scales of human immune investigation, ranging from molecular- to cohort-level investigation via immunology studies, and population-based investigation via epidemiology studies. **B**. Experimental immunology approaches: Experimental techniques are plotted based on the knowledge depth and breadth they can provide, and colored based on their category: systems immunology (blue) versus classical immunology (green). Generally, systems immunology provides a broader understanding due to high-throughput techniques. Some methods can be regarded as systems-level or classical approaches, depending on the experimental design, and are colored half blue and half green. **C**. Computational immunology approaches: Single-cell transcriptomics data illustrates how computational approaches enable the investigation of sex differences in immunity at multiple biological scales. DEGs, differentially expressed genes; PPI databases, protein-protein interaction databases, e.g. STRING [139]; TFBS databases, transcriptional factor binding site databases, e.g. JASPAR [140]; LRI databases, ligand-receptor interaction databases, e.g. CellPhoneDB [141], CellChatDB [142], and OmniPath [143]; GEMs, genome-scale metabolic models [144]. Created in BioRender. Cao, T. (2025) https://BioRender.com/7er4sm8.

Dissecting the contribution of different hormonal factors to human immune sex differences requires holistic profiling of immune dynamics while accounting for the complex modes of sex hormone regulation. Human immune function can be investigated at various levels: molecular, cellular, intercellular, organ, organism, cohort, and population (Fig. 3A) [121]. At the molecular level, genetic polymorphisms, variation in epigenomic landscape and in gene expression at transcription and translation levels regulate cellular states of different immune cell subsets, shaping their function. At the cellular level, individual immune subsets display varying phenotypes, specialized functions, numbers, and kinetics across human organs. Beyond the molecular and cellular levels, intercellular communication governs immune cell development, activation, effector functions, and tissue homeostasis, by coordinating signals both among immune cells and between immune and non-immune cells such as epithelial, stromal, and endothelial cells [122]. These intercellular interactions occur at the tissue level via direct cell-to-cell ligand-receptor contact or via soluble factors, including cytokines, chemokines, pathogen-associated molecular patterns, metabolites, and hormones. At the organ level, the magnitude of the network further increases compared with the intercellular communication within a niche. Coordinated interactions between immune and non-immune cells within the tissues ensure homeostasis and responses to infection/injury in an organ-specific manner. Investigating sex differences in human immunity at the organism level represents a complex challenge, as it requires integrating tissue-level immunity with systemic communication throughout the body. The understanding of sex differences in human immune function at a human cohort level enables the identification of patterns in immunity within a group of individuals that share a specific condition, which can be comparisons between males and females, or within the same individuals over time in longitudinal human cohorts, such as comparisons of varying levels of sex hormone over time (Fig. 2). Lastly, investigating sex differences in human immunity at the population level through epidemiological studies can highlight variability in the immune system across various demographic groups [123–125] and across environments [126, 127]. The exposome encompasses all environmental exposures throughout life, which are heavily conditioned by gender effects, such as microbiome composition, infection history, and lifestyle factors. Investigation of exposome influences poses as a great approach to assess how gender- and sex-specific exposures shape immunity [128]. In addition, deconstructing the variables underlying biological sex by focusing on genetic, hormonal, and environmental factors breaks through the binary restriction of the terms male/female and promotes mechanistic understanding and health equality across genders. In this context, systems immunology readily incorporates data-driven approaches and can aid in decoding immune sex-related variation in humans.

Classical immunology research has traditionally employed hypothesis-driven approaches to investigate human immune regulation by closely examining individual cell populations and molecular underpinnings one at a time. These molecular and cellular reductionist approaches, such as qPCR, Western blot, and ELISA, have provided critical knowledge depth into the functions of specific immune cell subsets, signaling pathways, and molecular mediators, establishing the foundation of our mechanistic understanding of the immune system (Fig. 3B). The current unprecedented development of high throughput technologies now supports a more systematic framework for investigation of immunity, significantly expanding the breadth of our knowledge in human immunology. Unlike reductionist approaches, systems immunology supports the simultaneous analysis of multiple immunological parameters. As a consequence, systems immunology (i) facilitates a holistic view of immune interactions and networks, (ii) provides mechanistic understanding across biological levels of regulation, (iii) enables the identification of novel biomarkers and (iv) supports the determination of immune heterogeneity within cohorts. This integration is made possible through a combination of diverse cutting-edge omics techniques and advanced computational methods. Such experimental techniques include profiling immune cell composition using cytometry techniques, analyzing genetic contributions to immune function via the genome through genome-wide association studies (GWAS), capturing immune cell gene expression profiles via microarrays, transcriptomics and epigenomics technologies, probing the immune proteome using proteomics methods such as mass spectrometry, Olink [129], and SomaScan [130], and finally employing CRISPR screens [131] and RNAi to dissect gene function and regulatory networks in immune responses (Fig. 3B). Conventionally, bulk omics measure the “averaged” omics profile of a pool of cells, limiting interpretation of results in heterogeneous samples, such as blood or tissue samples that present diverse immune cell type composition. Conversely, the burgeoning single-cell techniques provide unprecedented resolution of the immune system at single-cell resolution. Apart from the popular single-cell RNA sequencing (scRNA-seq) capturing the cellular transcriptome, single-cell techniques can be applied to different molecular modalities, including genomics and epigenomics [132], such as profiling chromatin accessibility via single-cell ATAC sequencing (scATAC-seq) [133] and mapping protein-DNA interactions with nano-CUT&Tag [134]. Alternatively, single-cell multiomics allows measuring of several molecular modalities of same group of cells simultaneously, with usual combinations of transcriptome with epigenome, proteome, and genomics [135], such as combining transcriptome and surface protein profiling in Cellular Indexing of Epitopes by Sequencing (CITE-seq), or coupling B Cell Receptor (BCR) [136] or T Cell Receptor (TCR) [137] sequencing with scRNA-seq in Single Cell Immune Profiling provided by 10X Genomics. Thus, systems immunology generates rich, multi-layered data that enables hypothesis generation and can reveal how biological sex regulates complex immune networks across biological scales, which can be further validated by traditional immunology studies [138].

Data generated from systems immunology studies are generally characterized by a high number of observations (e.g. samples, cells) and features (e.g. transcripts, proteins), requiring advanced computational methods for data statistical modelling and machine learning. Typical scRNA-seq analysis of immune sex differences includes determining immune subset composition differences, differential gene expression, and pathway analyses such as Gene Set Enrichment Analysis (GSEA) [139]. Advancing standard analysis by combining scRNA-seq data with external databases, metadata, and other omics layers enables a more mechanistic and holistic interpretation (Fig. 3C). For instance, gene regulatory networks can be inferred from scRNA-seq datasets, together with single-cell epigenomics and gene regulatory databases (e.g. protein-protein interaction databases [140] and transcription factor binding site databases [68, 141], revealing putative molecular mechanisms for biological sex regulation of the immune system. At the intercellular level, cell-cell communication networks can be inferred from transcriptomic and proteomic data using ligand-receptor interaction (LRI) databases [142–144], which provide insights into intercellular signaling shifts underlying sex differences in immunity. Moreover, integration with genome-scale metabolic models [145] can reveal sex differences in the metabolism of immune cells, with recent work demonstrating sex hormone biosynthesis in immune cells [146]. Additionally, supervised predictive modeling based on clinical records and multiomics data can improve biomarker discovery and stratification of patients for more precise medical care (Fig. 3C).

Below we discuss how systems immunology has been advancing our understanding of human sex differences in the healthy immune system at steady state, ranging from cell composition, transcriptomics, epigenomics, metabolism, cell-cell interactions, to plasma proteomics. Thereafter, we examine systems-level applications to investigating sex differences upon immune perturbations.

### Immune system at steady state

#### Cell composition

The immune system of a human being encompasses over one trillion cells throughout the body, with immune subset composition varying across tissues [147]. Immune cells are typically defined by surface and intracellular protein markers using flow, spectral, or mass cytometry. Flow cytometry identifies markers via fluorescently labelled antibodies, with cell classification being achieved by setting hierarchical thresholds. However, flow cytometry antibody panels can only incorporate up to 30 parameters due to significant overlap between fluorescent signals [148]. Spectral cytometry enhances flow cytometry capabilities by incorporating a fluorophore’s whole emission spectrum rather than specific wavelengths, allowing for an additional number of parameters [148]. Further, mass cytometry (CyTOF) is based on isotope-tagged antibodies and mass spectrometry-based quantification and enables the inclusion of over 60 parameters simultaneously [149]. Lack of standardization—due to variability in experimental protocols, such as choice of markers and antibodies, gating strategies, and analysis—limits reproducibility [150]. Initiatives such as Optimized Multicolor Immunofluorescence Panel (OMIP) aim to standardize experimental cytometry panels and protocols [151]. For instance, a 50-color spectral panel was developed for extensive immune phenotyping, focusing on T cells and dendritic cells [152]. Beyond experimental standardization, data-driven approaches can identify immune subsets from high-dimensional cytometry using methods such as dimensionality reduction (e.g. principal component analysis and uniform manifold approximation and projection) and unsupervised clustering [153,154], among other machine-learning tools [155]. For instance, Infinity Flow integrates traditional flow cytometry with a machine learning algorithm for simultaneous quantification of hundreds of proteins [156]. These advanced approaches provide powerful frameworks for studying cellular heterogeneity, such as sex differences in immune cell populations.

Biological sex impacts human immune cell composition. Females display a higher frequency of blood naïve CD4^+^ and CD8^+^ T cells [157], iNKT [158], NKT cells [157], NCR^+^ CD56^+^ ILC [157] and ILC3 [157], mucosa-associated invariant T (MAIT) cells [157], and plasma cells [159], while males have a higher frequency of blood myeloid cells, including monocytes [157, 159,160], basophils [160], neutrophils [160], and eosinophils [160], but also lymphoid cells such as NK cells [157, 159, 161] and γδ T cells [158]. Notably, the frequency of blood monocytes in females increases with age, eventually matching the increased percentages in males [162]. Additionally, a study with over 173,480 individuals showed that age, sex, and menopause status contributed to 40% of the variation in blood immune cell frequencies [163], highlighting the strong impact of biological sex in blood immune composition. Human immune sex differences in immune composition are not limited to the blood. Sex differences are observed in hematopoiesis, with male bone marrow progenitors producing red blood cells at higher rates, mostly due to testosterone [164]. Yet, research regarding sex differences in the development of human immune subsets in the bone marrow is lacking. Females display increased thymic output [165,166], which is an indicator of increased T cell maturation rate when compared to males. In addition, tonsils from female pediatric donors contain a higher frequency of memory B cells, while males show increased proportions of activated and naïve B cells [167].

Sex hormone changes throughout life impact human immune cell composition. Before puberty, males display increased CD4^+^ T regulatory counts in the blood, whereas females show increased counts of conventional CD4^+^ T cells [168]. During puberty, CD4^+^ and CD8^+^ memory T cells increase in both sexes, together with a male-specific decrease in CD19^+^ cells [169]. Females at reproductive age exhibit increased neutrophil frequencies, lower lymphocyte percentages, and higher neutrophil-to-lymphocyte ratio (NLR), a pattern that reverses after menopause [160]. While band neutrophil levels are higher in males versus females during reproductive age in healthy and autoimmune states, pregnancy increases band neutrophil signatures, which intensify with gestational age [170].

In addition, exogenous sex hormone therapy also impacts immune cell composition. For instance, MHT reduces the frequency of circulating plasmacytoid dendritic cells (pDCs), CD8^+^ MAIT cells and CD24^+^ CD8^+^ central memory T cells [171]. Further, high levels of estrogen during controlled ovarian stimulation during fertility treatment increases neutrophil, monocyte, and CD4^+^ T cell counts with a concomitant decrease in CD8^+^ T cells [172]. In contrast, androgen ablation using GnRH agonists in aged males increases lymphocyte levels, especially NK and T cells, the latter being attributed to increased thymic output [173]. Moreover, gonadotropins such as LH have been reported to expand bone marrow progenitors *in vitro* [82]. These studies showcase the complexity of biological sex effects, particularly through hormones, on the immune composition across tissues and highlight the need for comprehensive studies addressing biological sex effects in immune cell composition across human tissues.

#### Transcriptomics

While variation in immune cell composition has a prominent effect on immunity [174], elucidating cellular states and functional activity of immune cells is essential for a deeper comprehension of the mechanisms underlying immune sex differences. Microarrays and RNA sequencing (RNA-seq) are the two main techniques used to profile the bulk transcriptome, with microarrays quantifying the expression of a panel of predetermined sequences, while RNA-seq employs high-throughput sequencing of all or targeted sequences [175, 176]. These two technologies have been usefully employed to capture the transcriptomic differences in blood immune cells between males and females.

Through an integrated multi-cohort comparison of publicly available whole blood microarray datasets of 458 healthy individuals, Bongen *et al.* identified 144 differentially expressed genes (DEGs) between the sexes, including 94 female-biased and 50 male-biased genes [162]. Several of the DEGs are located on sex chromosomes, including 17 out of 25 X-linked genes that are known to escape from XCI (e.g. *XIST*, *RPS4X*, and *CD40LG*). This study also reported that 75% of sex-biased genes are located on autosomal chromosomes, indicating that sex differences in immunity at the transcriptome level extend beyond chromosome composition differences between the sexes [162] and suggest that other mechanisms—including hormonal effects—mediate sex differences. In fact, analysis of blood microarray data of 5,241 healthy individuals revealed that 7 out of 20 genes that have the highest fold change between sexes are strongly linked to estrogen regulation [177], including the G protein-coupled estrogen receptor *GPER*, and *ADM* [178] and *ERG* [179], both of which are ERα-regulated targets identified in rodents. Female-biased genes are enriched for autoimmune disease pathways, particularly RA gene sets [177], providing a perspective of how sex-biased gene expression may contribute to a higher prevalence of autoimmunity in females.

Further resolution of transcriptomic findings is revealed through single-cell sequencing methods, including plate-based [180, 181], droplet microfluidics-based [182], combinatorial indexing [183] scRNA-seq, among others. Huang *et al.* used scRNA-seq to analyze PBMCs from males and females across age groups, revealing that males have more aging-associated DEGs than females. Sex-associated DEGs are observed across all immune cells, increasing in number with age, with females showing upregulated T cell activation and B cell signaling pathways, and males exhibiting heightened innate immunity and inflammatory pathways in monocytes [159]. TCR [137] and BCR [136] sequencing at the single-cell level further enables investigation of sex differences in clonality and diversity of T and B cell repertoires. Sex differences regarding TCR diversity and clone size have been reported, as they are linked to the aforementioned decreased thymic output in males [165,166]. TCR repertoires in females are more diverse, while clonotypes with high frequencies are more common in males [184]. Further, HLA genes show sex-specific effects in driving the selection and expansion of T cells with specific TCRβ variable regions [185]. Similarly, although BCR repertoire diversity has been reported to be higher in females [186], it still warrants further examination [187]. In addition to single-cell sequencing alone, CITE-seq—typically used for clustering and identification of cell types—can be used to compare protein profiles across conditions [188–190], although sex differences have not yet been addressed this way.

Sex hormones significantly impact the human immune transcriptome. Sex differences at the bulk transcriptomic expression level in PBMCs decrease after menopause, but increase with hormonal contraceptive use, indicating the role of sex hormones in regulating PBMC gene expression [177]. Testosterone also regulates the immune transcriptome at steady-state, with testosterone therapy modulating the crossregulation of IFN and tumor necrosis factor (TNF) responses by decreasing IFNα response gene sets and increasing TNF signaling via NFκB following MHT [171]. Single-cell analyses also suggest a role for testosterone signaling inhibition in antitumor immunity, as CD8^+^ T cells that respond to systemic antiandrogen treatment have increased TCR and IFNγ signaling [116]. Combined, these studies suggest that sex hormones may directly regulate immune cell function via sex hormone receptors.

#### Epigenomics

Given their role in shaping gene expression, sex hormones likely drive immune sex differences at the gene regulatory level. Epigenomic methods provide insights from chromatin accessibility (e.g. DNase-seq [191], ATAC-seq [192], histone modification) and transcription factor regulation (e.g. ChIP-seq [193], CUT&RUN [194], CUT&Tag [195]), to chromosome interactions (e.g. Hi-C [196], HiChIP [197, 198]). Among these, ATAC-seq stands out as a widely used technique, which uses transposase enzymes to cut open regions of chromatin and insert adapters for sequencing of these regions. Exploring sex differences with both RNA- and ATAC-seq, Márquez *et al.* profiled females and males aged 22 to 93 years, revealing a shift from adaptive to innate immunity with age through concordant transcriptomic and epigenomic age-related decrease in T cell functions and increase in NK and CD8^+^ T cell cytotoxicity and monocyte function in both sexes [199]. Yet, aging also impacted the transcriptome and epigenome of immune subsets differently between the sexes, with male-specific decreases in B cell-related genes, alongside greater age-associated monocyte activation in males [199].

Single-cell epigenomics analysis via scATAC-seq shows that sex hormones have long-lasting effects on the human immune cell epigenomic state. One year of MHT increases transcript factor activity of all canonical NFκB pathways in T cells and NK cells [171]. Coupled with increased IFNγ production by NK cells pre-treated with androgens, these results may suggest an NFκB-mediated regulation of lymphocytic function following testosterone treatment [171]. Yet, while single-cell epigenomics techniques provide unprecedented resolution and analytical tools are available, studies addressing epigenomic differences between males and females in immunity remain surprisingly limited. The availability of single-cell chromatin accessibility atlases [200, 201] provides a unique opportunity to explore general sex differences in the immune cell epigenomic landscape using existing resources.

#### Metabolomics

Immune responses are energetically intensive and require coordinated metabolic adaptations both at the cellular and systemic levels. Immunometabolism explores how metabolic processes influence immune cell function, emerging as a promising layer in systems immunology by adding a new dimension to the complexity of immune responses [202]. Immunometabolism relies on approaches based on mass spectrometry, which imply measuring the concentrations of metabolites (e.g. lipids, amino acids) either inside of cells, or in extracellular fluids like plasma. For measuring *in vitro* cellular metabolism, Seahorse assays enable real-time evaluation of immune sex differences by quantifying glycolysis, mitochondrial respiration, and substrate use, providing mechanistic insights such as the balance between glycolysis and fatty acid oxidation [203]. In contrast, cytometry- or sequencing-based techniques enable inference of metabolic activity at single-cell resolution [202]. SCENITH combines specific enzyme inhibitors and protein synthesis to assess the reliance on specific metabolic pathways (e.g. glycolysis, oxidative phosphorylation) via flow cytometry across immune subsets [204]. Similarly, Met-Flow [205] enables flow-cytometry detection of metabolic enzymes and transporters (e.g. lactate dehydrogenase, glucose transporters), thus providing a static snapshot of metabolic-protein expression. Alternatively, metabolic states can be inferred from scRNA-seq data combined with genome-scale metabolic models [145] utilizing computational approaches such as METAFlux [206] or Compass [207].

The human metabolome is highly different between the sexes, evident in both health [208] and disease [209, 210]. While sex differences in mitochondrial biology across organs have been identified, such as increased mitochondrial content in female leukocytes [211], direct exploration of sex differences in immunometabolism has only recently begun [30, 212]. Macrophages from placentas of females have an increased dependency on fatty acid oxidation, whereas those from males show increased glycolytic activity, correlating with the capacity to remove apoptotic cells [213]. Blood CD4^+^ T cells derived from female severe asthma patients are more dependent on glutamine uptake than those from males [214]. Sex differences in immunometabolism are likely to be driven by sex hormones, which are known to modulate systemic metabolism. For instance, estrogen promotes fat storage in subcutaneous tissues and glucose uptake by skeletal muscle, whereas testosterone enhances protein synthesis and lipolysis [215]. At the cellular level, male neutrophils exhibit increased mitochondrial respiration under stress compared to female macrophages, which correlates with decreased responsiveness and IFNγ responses and is reversed upon estradiol treatment [216], highlighting the emerging role of sex hormones in regulating immunometabolism in humans.

#### Spatial omics

Spatial omics enables molecular analysis while preserving tissue structure, adding location information that other omics lack [217]. Visium [218] uses a chip covered by barcoded poly-A probes to capture mRNA in the tissue samples, offering an exploratory way to map gene expression. Hybridization-based *in situ* sequencing (HybISS) [219] uses padlock probes and rolling circle amplification to detect hundreds of genes in a single tissue section. Compared to Visium, HybISS is less suited for exploratory analyses, focusing more on targeted specific genes or pathways rather than broad transcriptome profiling. However, it provides subcellular resolution, which is not currently achievable with Visium. Deterministic barcoding in tissue for spatial omics sequencing (DBiT-seq) [220] is an open protocol for spatial omics that enables exploratory examination. It employs a different strategy to barcode the spatial information before sequencing, based on microfluidics, which theoretically leads to higher resolution compared to Visium. In addition, spatial proteomics couples imaging techniques with targeted antibody-based approaches, to determine protein expression within tissues. Methods like Phenocycler [221] (previously named CODEX) and COMET [222] allow the study of protein distribution in tissue samples. Both use multiplexed immunofluorescence with up to dozens of markers (maximum 60-plex for CODEX [221] and 40-plex for COMET [222]), offering flexibility through pre-defined or custom antibodies [223], enabling the exploration and biological interpretation of protein abundance and location, and facilitating the examination of cellular and molecular information in specific tissues.

Spatial omics can offer unique insights by preserving spatial context, allowing for the investigation of sex differences in immunity within the tissue environment. Recently, Stankiewicz *et al.* utilized spatial omics with a 48 antibody Phenocycler panel to investigate the thymic architecture in 4- to 5-month-old males and females [224]. This approach enabled the detection of higher double-positive T cells and cortical epithelial cells in females and higher single-positive T cells and medullary thymic epithelial cells in males, suggesting a female bias toward positive selection and a male bias toward negative selection [224]. Sex hormone regulation of breast immune cells has been recently shown in transgender men undergoing MHT using Phenocycler [225]. Androgen treatment leads to changes in cellular distribution, with a decrease in macrophages and an increase in CD4^+^ T cells close to the epithelium. Functional changes are also observed, including lower antigen presentation activity of macrophages and enriched activation pathways (e.g. TCF7) in CD4^+^ T cells. In addition, fibroblasts near the epithelial niche express higher IL-16 upon MHT, potentially explaining the increased T cell infiltration [225]. These studies highlight how spatial omics can shape a new avenue of immune exploration by determining the distribution, function, and crosstalk of tissue immune cells between the sexes.

#### Cell-cell communication

Immune responses emerge from a network of interactions among cells that can be of direct contact or through soluble factors such as cytokines, chemokines, etc. Cell-to-cell interactions are commonly inferred from LRI databases [142, 143]. In such, LRI are established from experimental studies utilizing electron or optical microscopy, chemical tagging or mechanical force assays, and further validated by co-immunoprecipitation and functional studies [226]. Cell-cell interactions can be evaluated by combining LRI databases, such as CellPhoneDB [142] and CellChatDB [143], with single-cell or spatial transcriptomics data through a multitude of computational approaches, such as CellChat [143] and MultiNicheNet [227] (reviewed in ref [228]). Other tools, such as DIALOGUE [229], have been developed to identify novel multi-cellular programs (i.e. sets of coordinated gene activity across various cell types) by evaluating correlations in gene expression levels from single-cell and spatial transcriptomics data. Further combination of the identified multi-cellular programs with external LRI databases can reveal potential regulatory mediators.

Approaches assessing cell-cell interactions have uncovered sex and age differences in cell communication in PBMCs [159]. Young females show increased BAFF and APRIL signaling from conventional dendritic cells type 2 (cDC2) to plasma cells and memory B cells [159], which have been reported to be dysregulated in autoimmune disease [230]. Further, chemokine signaling is increased with aging in males versus females, such as IL-1B–IL1R2 from monocytes to monocytes, and CXCL10–CXCR3 from DCs to T cells [159]. Sex hormones also regulate human PBMC cell-cell communication. MHT leads to further upregulation of IL-6, TNF, IL-15, and IL-12B production by monocytes following LPS stimulation, which in turn trigger NK and CD8^+^ T cells to activate genes related to cytotoxicity, such as *SOCS1* and *RUNX2* in CD8^+^ T cells, and *IFNG* and *GZMB* in NK cells [171]. Together, these studies showcase that immune sex differences extend beyond intracellular mechanisms; even small differences within specific cell types may be amplified through cell–cell communication networks, with biological sex influencing intercellular signaling and contributing to broader differences in immune responses and related diseases.

#### Plasma proteomics

Immune plasma proteomics enables the comprehensive study of soluble factors in the blood and provides a snapshot of a person’s systemic immune environment. Although mass spectrometry-based proteomics allows for untargeted proteome profiling with high resolution, it can lack sensitivity to low-abundance proteins without prior enrichment, such as cytokines or transcription factors [231]. On the other hand, proximity-based extensions assay [129], and aptamers-based technologies [130] provide simultaneous measurements of thousands of proteins at a time from samples of only a few microliters. However, they are limited by the selected panel of antibodies or aptamers chosen for the detection and their specificity regarding isoforms or protein complexes. For instance, Olink has a 92-protein panel with lowly abundant proteins involved in inflammation [232]. Notably, males have been reported to have increased concentrations of 31 of these inflammatory proteins in healthy state, including TNF family members (TRANCE, TRAIL), CCL11, and MCP-2, whereas females only showed increased concentrations of 3 proteins, OPG, and myeloid cell regulating factors CSF-1 and Flt3L [233]. Immune plasma proteomics is also impacted by exogenous sex hormone therapy. Olink proteomics analysis shows that TNF family proteins (TNF, RANKL, TNFSFR9, and TRAIL) are also increased in plasma following MHT [171]. Yet, cisgender men still exhibit higher TNF levels when compared to transgender men and transgender women [234]. Further, the TNF family member that regulates B cell function, BAFF, is higher in both transgender and cisgender women, indicating a role for estrogen regulation. Moreover, use of oral contraceptives has profound effects on the plasma proteome of females [235] and suppresses the levels of many inflammatory proteins such as TNF family members TRANCE and TWEAK, and CCL11 [161]. Altogether, integration of plasma proteomics with other systems-level methods will be key to enable the determination of the cellular and tissue origins of the sex differences in immune plasma proteome in humans.

### Immune sex differences upon immune perturbation

The human immune system is highly dynamic. Thus, sex differences are not only manifested at steady state, but are also evident across perturbed immune states, such as upon infections [4,5] and vaccination [13,14]. Sex differences upon perturbation can be explored through *in vitro* and *ex vivo* studies of PBMC, whole blood, tissue sections, and organoids. However, controlled *in vivo* studies offer a more comprehensive framework by enabling the identification of system-level responses that can be directly linked to clinical outcomes.

#### Blood-stimulation models

Studying immune responses under controlled conditions typically involves direct *ex vivo* whole blood stimulation, which introduces less technical biases and preserves the physiological context of the immune response, or *in vitro* stimulated PBMCs, with increased experimental flexibility and mechanistic insights [236]. To this end, whole microbes (e.g. bacteria, viruses, fungi), antigens derived from them, or synthetic agonists (e.g. TLR agonists) are used to analyze specific immune responses via modulation of proliferation, gene expression, or cytokine production. For instance, after exposing whole blood from male and female donors to bacterial, viral, and fungal agents *ex vivo*, 181 genes were reported to show sex differences only after stimulation [237]. Further, females consistently overexpressed genes across all stimulations [237], altogether indicating that these sex-specific effects were mostly shared across stimuli. For example, females show higher expression of *ICOS*, which negatively influences T cell activation, while males have stronger expression of *CD14*, involved in LPS sensing [237]. In contrast, males produce more monocyte-derived cytokines after LPS stimulation of whole blood and PBMC exposure to *Candida albicans conidia*, while females produce more Th17-related responses to *C. albicans hyphae* [238], suggesting stimuli-dependent sex-specific responses.

Sex hormones also regulate immune responses assessed *in vitro*. Oral contraceptive usage reduces female IFNγ and TNFα production after LPS whole-blood stimulation [238]. Whole-blood LPS stimulation also induces higher TNF in cisgender and transgender men, compared to transgender and cisgender women [234], suggesting a role for sex hormone rather than sex chromosome regulation in higher TNF responses in males. Additionally, higher testosterone in females was associated with stronger IFNα responses after stimulation with R848, a TLR7 agonist, whereas increased testosterone correlated with lower IFNα responses in males [239]. Altogether, blood stimulation studies underscore how the reactive and dynamic nature of the immune system amplifies steady-state differences between the sexes—aligning with the observed sex differences in infectious and immune-related diseases—and how the sex hormone milieu plays a prominent role in shaping immune responses.

#### Tissue-stimulation models

Tissue samples, such as tumor slices or tonsil organoids, can be used to evaluate sex differences in immune responses in a more *in vivo*-like tissue microenvironment. These tissues can readily be obtained from diverse sources, including discarded materials from deceased individuals (e.g. autopsies, organ donors) or living individuals (e.g. clinical biopsies, surgery) [240]. Tumor tissue is used in personalized immunotherapy research, as it enables direct evaluation of a treatment in individual patient tumors [9]. Although biological sex is relevant in cancer immunotherapy [241–243], to date no studies have focused on assessing sex differences through *ex vivo* tumor stimulation assays. Besides, tonsil organoids offer unique advantages to simulate adaptive immune responses *ex vivo*, such as preserving the *in vivo* lymphoid tissue architecture, rich in B and T cells, and showcasing extensive use in vaccine research [167, 244, 245]. For example, tonsil organoids from pediatric donors exposed to live-attenuated influenza vaccine (LAIV) showed sex-dimorphic responses, with females exhibiting increased specific antibody responses even though male-derived organoids contained more activated B cell subsets after 7 days [167]. Future exploration of tissue-stimulation models will provide a deeper understanding of mechanisms underlying immune sex differences in more realistic environments that encompass tissue-resident immune cells and other local factors.

#### *In vivo* immune perturbation following infection

Longitudinal sampling of human cohorts enables investigation of biological sex effects following perturbations (e.g. infection, vaccination) while controlling for the considerable human interindividual variability. Immune responses after infection can be explored at different levels (Fig. 3A). Temporal dynamics of plasma proteins vary between males and females with severe COVID-19. While females show higher CCL26 and lower IL3RA shortly after symptom onset compared to males, they exhibit similar trends in expression over the course of recovery. In contrast, IL1RL2 decreases to normal levels faster in females following recovery [246]. Male patients hospitalized for COVID-19 show increased CCL5 [247], which has been associated with COVID-19 disease severity [248]. In the same study, flow cytometry of PBMCs identified higher counts of non-classical monocytes (CD14^+^ CD16^+^) in male patients compared to females [247]. Moreover, females have increased BCR clonotype size and more BCR mutations compared to males during COVID-19 infection [189]. In addition, a series of studies have reported sex differences regarding long COVID patients and patients who quickly recovered after SARS-CoV-2 infection with no long-lasting sequelae [18, 249–251]. During acute infection, males who developed LC show decreased monocyte and DC frequencies and higher NK counts, while females who develop LC show increased exhausted T cells and cytokine-secreting T cells [251]. Moreover, CD14^+^ monocytes from males who later developed LC displayed impaired CD40LG-CD40 signaling [249], which is important for T cell and monocyte interactions during inflammatory responses, and increased pro-inflammatory IFNγ and IL-15 [249], and TGF-β [250] signaling. Of note, these findings are concordant with elevated TGF-β-family members and IL-8 in serum plasma of LC males [251]. In contrast, CD14^+^ monocytes in females showed increased CCL5 signaling from T and NK cells [249], coupled with lower *TGFB1* expression [250].

Serum Epitope Repertoire Analysis (SERA) enables profiling of peptide-specific antibodies, facilitating exposome analysis through detection of previous exposure to pathogens [252] or autoantigens associated with autoimmune diseases [253], thus providing insights into an individual’s immune history. Similarly, VirScan systematically profiles existing immunity against viruses based on phage display technology [254]. SERA analysis reveals that females with LC are overwhelmingly seropositive to HSV-2 [251], which has been linked to increased risk of post-acute infection syndromes [18], and showcase sex-specific disease association with immune histories. Notably, a pre-print study recently highlighted a role for sex hormones in LC [251], where female testosterone is negatively associated with LC risk and symptom burden, while male estradiol levels have a protective effect against LC symptom severity [251]. Moreover, when whole blood from LC patients is stimulated *ex vivo* with herpesviruses antigens, females show increased antibody reactivity to EBV, HSV-2, and CMV. Interestingly, low testosterone is associated with increased reactivity to EBV and HSV-2 viruses in both females and males, and to CMV only in females with LC [251]. Altogether, investigating sex differences within the context of infectious and infection-related syndromes can further validate regulatory mechanisms underlying sex differences in immune responses in an *in vivo* setting.

#### *In vivo* immune perturbation following vaccination

Vaccination poses an ideal framework to study *in vivo* immune responses in a controlled environment, while holding special relevance for identifying sex differences in disease management. In fact, evidence from aged adults (60–80 yrs) supports a female advantage early after influenza vaccination. Females upregulate type I IFN and classical-complement activation genes at 3 days [255] and show increased numbers of influenza-specific memory B cells 28 days after vaccination, linked to increased expression of B cell gene modules, whereas males exhibit higher NK and cytotoxic T cell gene expression at day 3 and 28 [256] and increased complement and protein processing pathways at 28 days [255]. In addition, influenza vaccine responses can be impacted by previous mild COVID-19 disease [257]. Before vaccination, COVID-19 recovered males have lower innate immune gene expression signatures in monocytes and increased CD8^+^ T cell activation signatures compared to unexposed males and recovered females. Shortly after vaccination, recovered males display increased frequencies of and higher IFNγ production by a subset of T cells (GPR56^+^ CD8^+^) due to a simultaneous increase in IL-15-producing classical monocytes, while such an effect is absent in females. Interestingly, these differences between the recovered and unexposed cohorts fade at 28 days after vaccination [257].

Sex hormones may explain sex differences observed after vaccination. The role of sex hormones was evaluated in adolescents in responses after LAIV and SARS-CoV-2 mRNA BNT162b2 covaccination [258]. At baseline, males show lower numbers of pDCs in blood, and free testosterone is negatively associated with the production of type I interferon (IFN-I) by pDCs. Conversely, males’ pDC IFN-I production increases and reaches comparable levels with females’ one day after vaccination. Surprisingly, DHT is associated with elevated IgG titers, and men show higher antibody responses after co-vaccination with LAIV and BNR162b2 [258]. These findings are concordant with the observation that previous COVID-19 infection enhances influenza vaccine response in males [257]. Females also show elevated antibody responses and expression of cytokines in the serum following influenza vaccine, while males with higher testosterone concentrations show the lowest antibody responses [259]. Furthermore, Olink profiling of 92 inflammatory proteins revealed that several cytokines, including CXCL1 and IL-18, are negatively correlated with blood testosterone concentration after BCG vaccination [233], showcasing the *in vivo* immunosuppressive role of testosterone following vaccination. In contrast, no significant changes in protein levels were observed in females, though female PBMC stimulation *in vitro* with *M. tuberculosis* show increased IFNγ production when compared to males [233]. These studies emphasize the complexity of the interplay of sex hormones and immune responses to stimuli when investigated *in vivo* or *in vitro*, and—beyond the male/female binary—they highlight that variation in immunological parameters can be dependent on the relative concentrations of sex hormones.

#### *In vivo* immune investigation across tissues

Investigating immune sex differences across tissues is crucial for understanding disease susceptibility, symptomatology, severity, and mortality [260, 261] and modeling disease comprehensively [30, 240]. Systems immunology can accommodate multi-tissue modeling, moving beyond traditional reductionist methods that focus on individual tissues at a time. For instance, this has been successfully applied to survey androgen effects across tissues in mice [262]. Such holistic approaches can identify networks of interactions between cells and molecules across organs [261]. Recently, comprehensive human datasets spanning different organs are being generated, such as from Domínguez Conde *et al.*, which contains data from immune cells from 12 different organs [263], and from Suo *et al.* which profiles the developing immune system across organs [264]. In disease, male COVID-19 patients show increased interactions between macrophages and T cells in nasal tissue across mild and severe cases compared to females. In parallel, only PBMCs from severe cases in males display elevated pro-inflammatory macrophage activation (TLR/cytokine gene sets) and heightened macrophage-CD8^+^ T cell interactions [265]. In sum, while tissue differences might explain sex-linked susceptibility to COVID-19, blood disparities reflect systemic alterations that likely underlie higher male mortality.

Overall, systems immunology studies employ experimental design, omics techniques, and computational approaches that are complementary to classical immunology studies, and underline the importance of comprehensive studies in profiling sex differences in the immune systems across organs for improved understanding of disease pathogenesis and immune kinetics.

## Future directions

### Multiomics integration

To better understand the detailed impact of sex on immune system, multiomics integration is an inevitable future direction. While diseases often result from disruptions in intricate pathways across omics layers (genomics, transcriptomics, proteomics, and metabolomics), single-omics studies cannot capture the essential cross-layer interactions. Multiomics integration offers a solution to this challenge and can be categorized into two types. Vertical integration is applied when using multiomics techniques to collect multi-modalities from the same samples/cells simultaneously, by using shared sample or cell identifiers to directly align each layer. Diagonal integration merges different omics modalities obtained from their respective mono-omics techniques (such as integrating scRNA-seq and scATAC-seq datasets), requiring advanced mapping techniques to link samples obtained from different assays [266, 267].

Multiomics integration offers a holistic perspective that extends the ability to identify sample/cell subgroups, discover biomarkers, and reveal potential molecular mechanisms underlying sex differences in immunity. Omics data is high-dimensional and noisy [268], making dimensionality reduction crucial before analysis. Instead of applying such preprocessing separately, joint approaches like joint non-negative matrix factorization (jNMF) [269,270] and multiomics factor analysis (MOFA [271] and MOFA+ [272]) are particularly useful to identify shared and omics-specific patterns via uncovering latent structures that reveal relationships between datasets. Additionally, feature selection tools such as mixOmics [273] support biomarker identification across layers, while regulatory network methods like SCENIC+ [274] integrate chromatin accessibility with transcriptomics to infer gene regulatory networks. Moreover, tracking of sex-specific immune changes via multiomics profiling over time—due to immune or sex hormone perturbations—requires analytic platforms designed for longitudinal multiomics data. PALMO (Platform for analyzing longitudinal multiomics data) [275] and Stabl [276 ] both provide frameworks to analyze time-course data across multiple omics layers, which can facilitate the investigation of immune sex differences and immune impact from sex across omics modalities in future studies. For instance, Sauerwald *et al.* investigated how immune states at baseline contribute to sex differences in SARS-CoV-2 infection outcomes by integrating whole blood bulk RNA-seq and serum Olink proteomic data from a cohort of young adults [277]. This multiomics integration analysis demonstrated that the pre-existing higher expression of interferon-stimulated genes in female neutrophils causally contributes to the female-bias antiviral responses, as reflected in molecular responses, viral load, and clinical symptoms [277], highlighting the potential of multiomics integration in revealing mechanisms underlying sex differences in human immunity.

### Addressing biological sex in analyses and prediction

While sex differences in immunity and disease are widely acknowledged, biological sex has historically been ignored in research [30]. Several bottlenecks prevent the inclusion of sex and gender considerations from basic science to clinical practice in immunology, ranging from aspects regarding study design and analysis to systemic discrimination and the lack of inclusion of vulnerable groups [278]. Even today, sex-disaggregated data is often not readily available. For instance, over 25% of publications in human immunology research up to 2009 did not specify the sex of the subjects [279]. In research studies where both sexes are included, often results are still not disaggregated based on sex, despite encouragement from guidelines such as the Sex and Gender Equity in Research (SAGER) [280, 281]. Tools have been developed to overcome some of these barriers using sequencing data. XYalign [282] addresses misalignment issues caused by X-Y chromosome homology by incorporating sex chromosome complement during alignment, improving accuracy in chromosomal sex identification of samples. Additionally, to address unspecified sex of the subjects, packages like SexInferences (https://github.com/SexChrLab/SexInference) and CellXY (https://github.com/phipsonlab/cellXY) have been developed to infer biological sex of the samples from either DNA sequencing, RNA sequencing, or scRNA-seq data. Large-scale sex label annotation of more than 450,000 human transcriptome samples was achieved by Flynn *et al*. [283], indicating a promising future direction that includes sex inference function into public sequencing repositories (e.g. GEO), enabling suggested sex annotation for each dataset.

Further, even when studies report sex differences, they often lack robust statistical support [284] as sex is frequently treated as a confounder rather than a relevant factor in analysis. However, designing studies with appropriate sample sizes to support meaningful comparisons between sexes is usually feasible [285]. Rather than only deriving findings representative of the average population by adjusting statistical models for sex, future studies should directly examine sex differences to generate more precise and applicable insights for both sexes. This approach is exemplified in a long COVID study, where inclusion of sex in analyses and prediction revealed sex-specific LC predictors, such as higher NK cells, decreased monocytes, and higher level of TGF-β for males, and increased cytokine-secreting T cells and higher EBV reactivity for females [251]. Building on this, incorporating omics data into machine learning models will reveal novel predictors, such as sets of genes or metabolites. However, limited research studies of sex differences in immunity have explored prediction models with omics data. One barrier to such inclusion is the limited samples compared to thousands of features measured from omics techniques, which tend to overfit the trained prediction models and thus lack generalization to other cohorts. In recent years, methods such as Stabl [276] have been designed to cope with such challenges. By noise injection followed by subsampling iterations, Stabl ensures a robust feature selection threshold that limits the number of features in the final model.

Analyses that disentangle biological sex effects on the immune system require comprehensively curated gene lists, including sex-biased immune modules, XCI escapees, and hormone-responsive genes across immune cell types. Systematic evaluation of sex-biased expression across tissues (e.g. Oliva *et al.* [286]), and identification of sex-specific expression signatures in immune cells (e.g. Bongen *et al.* [162]) enable the quantification of sex differences across disease states and lifespan. To distinguish sex chromosome from sex hormone effects, a key priority is identifying genes that escape X chromosome inactivation across immune cell types and activation states [35, 37, 287]. In addition, genome-wide identification of hormone-responsive genes, such as genes directly bound by nuclear hormone receptors or downstream of membrane-bound receptors (Table 1), is especially lacking in primary immune cells, as current data are mostly limited to cancer cell lines [70, 288, 289].

Importantly, neglecting biological sex in research has had consequences in medicine. For example, eight out of 10 prescription drugs withdrawn from the market between 1997 and 2001 in the US had greater undesired effects for women, even after equal prescription rates [290]. Moreover, the exclusion of pregnant individuals and other minorities from clinical trials has led to harmful therapeutic approaches and a lack of knowledge on how sex hormones impact health [278], highlighting the need to include women and minoritized populations in research. Lastly, sexism and other types of discrimination also have conditioned the amount of funding or research interest in sex differences [278]. Inclusion of gender-diverse and intersex cohorts can reveal immune signatures that do not neatly align with chromosomal or hormonal extremes [291], and highlight the need for sex-aware research moving forward to enable more personalized medical interventions.

### Open science’s role in the study of sex differences

Systems immunology heavily relies on experimental methods that produce large amounts of complex data, and requires data-driven analytical approaches. Thus, open science is at the core of systems immunology’s success and particularly critical for the robust study of sex differences. Open science refers to a set of practices aimed at making research reproducible and accessible. This involves several aspects such as utilizing accessible and reproducible methods for data analysis (i.e. code, software), data sharing, having public access to research results, and educational resources [292]. Adhering to the FAIR (Findability, Accessibility, Interoperability, Reusability) principles is vital for the development and future of human immunology research [293]. In this context, the study of sex differences in the immune system can greatly benefit from researchers adhering to such practices. First, data and its corresponding metadata, such as sex and gender labels, should be findable by hosting the data in adequate repositories and unique identifiers like DOIs (Table 2). Second, data should be accessible, prioritizing open access, allowing for automated access, and with appropriate ethical compliance for sensitive data, allowing for systematic evaluation of sex differences. Third, data should be interoperable and follow standard guidelines for readily integration among datatypes, which is essential for defining sex-linked immune signatures. Last, data should be reusable and thus include rich metadata detailing origin, purpose, and conditions for reuse [293]. Individual researchers and consortiums building reference datasets must include key metadata (i.e. sex and other demographic or biological factors) and ensure that the datasets reflect population-level diversity. For instance, sex annotation compliance across selected repositories relevant to systems immunology shows a widespread lack of clear distinction between sex and gender in the metadata fields (Table 2).

**Table 2.** Sex annotation compliance across selected repositories relevant for systems immunology. For each database, four aspects of sex metadata are assessed: (1) availability of sex metadata (**✓**: metadata field available, !: field not present, **x**: no option available), (2) requirement for submitting sex metadata (**✓**: required, !: recommended, **x**: not mentioned in guidelines), (3) clear distinction between sex and gender (**✓**: present, !: two categories but unclear definitions, **x**: no distinction), (4): use of controlled terminology (**✓**: required, !: recommended, **x**: free text).

Datatype	Databases	Sex metadata available	Sex metadata required	Sex vs. gender distinction	Uses controlled terminology
General	ImmPort	**✓**	**x**	**x**	**✓**
	EGA (European Genome-Phenome Archive)	**✓**	**✓**	**x**	**✓**
	BioSamples	**✓**	**✓**	**✓**	**✓**
Flow cytometry	FlowRepository	**✓**	**!**	**x**	**✓**
Transcriptomics	GEO (Gene Expression Omnibus)	**✓**	**!**	**x**	**x**
	GTEx (Genotype-Tissue Expression)	**✓**	**✓**	**x**	**✓**
Single cell transcriptomics	Single Cell Expression Atlas	**✓**	**!**	**x**	**✓**
	HCA (Human Cell Atlas)	**✓**	**✓**	**✓**	**✓**
Epitopes	IEDB (Immune Epitope Database)	**✓**	**!**	**x**	**✓**
Epigenomics	ENCODE (Encyclopedia of DNA Elements)	**✓**	**!**	**x**	**✓**
Proteomics	PRIDE (PRoteomics IDEntifications Database)	**✓**	**x**	**x**	**✓**
Metabolomics	MetaboLights	**✓**	**!**	**x**	**✓**

To advance systems immunology studies of immune responses across sexes, public data repositories, and consortiums generating comprehensive population-level reference datasets will play a central role. Deep profiling at population-scale provides a platform to discover robust differences between the sexes. The UK Biobank project is collecting genomics [294], plasma proteomic [295], among other data, all of which are linked to health registries in the UK. It has provided valuable insights into our understanding of human variation in the immune system related to sex, such as during COVID-19 infection [246]. In Sweden, initiatives such as Swedish CArdioPulmonary BioImage Study [296] or EpiHealth [297] have also included omics profiling in their studies and are predicted to elevate our understanding of immune variation at the population level. Together, open science resources and initiatives enable a deeper understanding of immune sex differences by providing the data and tools necessary for uncovering molecular, cellular, and systemic mechanisms underlying sex differences in immune responses.

## Conclusion

Sex differences are a fundamental aspect of human immunity. Advances in systems immunology pave the way for a more holistic understanding of how biological sex shapes the immune system in steady state and upon perturbation. As high-throughput technologies and computational tools become more accessible, adherence to open science principles is essential for systematic analysis of multimodal data. Population-level profiling paired with cross-cohort comparisons will enable the disentanglement of sex chromosome and sex hormone effects at all levels of immune variation. These efforts lay the foundations for personalized approaches to treating and managing diseases. Ultimately, this will ensure that differences between males and females and beyond the binary are properly accounted for in immunological research and clinical practice.

## Data Availability

No original data have been generated for this review.
